# Still at It—The N. Y. Medical Record

**Published:** 1887-09

**Authors:** 


Still at it.—Friends of Wm. Wood & Co., of whom there are many in the South and West, who admire their enterprise, pluck and liberality in dealing, had sincerely hoped that they, having seen the great extent to which the editorial course of the *New York Medical Record* with especial reference to the Congress, had alienated Southern and Western physicians, had muzzled the editor; and hence many in those sections read with surprise, and a feeling akin to disgust, the unjust and untrue stricture on the Congress in last issue of that publication. It "damns with faint praise" indeed; and still snarls and snaps, as in days of organization. The *Record* still sticks to it that the Congress was not "representative," but was made up of "Western men, with more from the South, a few from the East, and a sprinkling of Europeans;" and in face of a *success*, demonstrated by the *Record's* own publication of the many and excellent papers, by distinguished Europeans—pronounces it a failure—except as to numbers!

